# An Improved Channel Estimation Technique for IEEE 802.11p Standard in Vehicular Communications

**DOI:** 10.3390/s19010098

**Published:** 2018-12-28

**Authors:** Tong Wang, Azhar Hussain, Yue Cao, Sangirov Gulomjon

**Affiliations:** 1 College of Information and Communication Engineering, Harbin Engineering University, Harbin 150001, China; wangtong@hrbeu.edu.cn; 2 Department of Computer and Information Sciences, Northumbria University, Newcastle upon Tyne NE1 8ST, UK; 3 Nokia Shanghai Bell Innovative Park, Hangzhou 310014, China; gulomjon.sangirov@nokia-sbell.com

**Keywords:** IEEE 802.11p, Vehicular Networks, Intelligent Transportation Systems, constructed data pilots, vehicle-to-vehicle

## Abstract

IEEE 802.11p based Dedicated Short-Range Communication (DSRC) is considered a potential wireless technology to enable transportation safety and traffic efficiency. A major challenge in the development of IEEE 802.11p technology is ensuring communication reliability in highly dynamic Vehicle-to-Vehicle (V2V) environments. The design of IEEE 802.11p does not have a sufficient number of training symbols in the time domain and pilot carriers in the frequency domain to enable accurate estimation of rapidly varying V2V channels. The channel estimation of IEEE 802.11p is preamble based, which cannot guarantee a suitable equalization in urban and highway scenarios, especially for longer length data packets. This limitation has been investigated by some research works, which suggest that one major challenge is determining an accurate means of updating channel estimate over the course of packet length while adhering to the standard. The motivation behind this article is to overcome this challenge. We have proposed an improved Constructed Data Pilot (iCDP) scheme which adheres to the standard and constructs data pilots by considering the correlation characteristics between adjacent data symbols in time domain and adjacent subcarriers in frequency domain. It is in contrast to previous schemes which considered the correlation in the time domain. The results have shown that the proposed scheme performs better than previous schemes in terms of bit error rate (BER) and root-mean-square error (RMSE).

## 1. Introduction

Dedicated Short-Range Communication (DSRC) is considered a promising short-range wireless communication standard for vehicular communications [1]. Among other benefits, DSRC can provide cooperative driving-safety using Vehicle-to-Vehicle (V2V) communication. Various DSRC applications include: Cooperative forward collision warning [2], impending road hazards, an upcoming traffic jam, assistance in adverse weather, blind spot warning, traffic light optimal speed advisory, and remote wireless diagnosis [3]. However, ensuring communication reliability is very important for these mission-critical applications under highly dynamic V2V communication channels. The physical (PHY) layer design of DSRC system is inherited from the IEEE 802.11a standard, by reducing the signal bandwidth from 20 MHz to 10 MHz and operating frequency to 5.9 GHz. The main reason behind this inheritance is to reduce the manufacturing cost of the DSRC devices by making slight changes in the 802.11a based systems, which are readily available in market.

The 802.11a standard was originally developed for relatively stationary indoor environments. However, V2V wireless channel is extremely challenging for signal propagation due to: (1 Vehicular mobility which leads to a short channel coherence time and (2 the presence of mobile and stationary scatterers e.g., other vehicles and buildings, which results in a narrow coherence bandwidth. Recent channel sounding measurements have indicated that the coherence bandwidth is about 410 to 820 KHz and coherence time is about 0.3 to 1.0 ms in V2V channels, which are in direct contrast to the static indoor environments (a coherence time of 25 ms and coherence bandwidth of 1 to 3 MHz) [4]. The performance of the IEEE 802.11p system would significantly suffer due to degradation of transmitted signal waveforms in V2V environments. As a well-known fact, channel estimation plays a vital role in the design of any wireless communication system. A precisely estimated channel response (CR) is critical for the follow-up equalization, demodulation, and decoding [5]. Generally, the accuracy of channel estimation decides the reliability of wireless communication system. Therefore, in the V2V environment, the primary challenge is to determine accurate means of updating the channel estimate over the course of a packet length while adhering to the standard.

Besides IEEE 802.11p, the millimeter-wave communication standard IEEE 802.11ad is also emerging as a preferred near-field communication system for V2V. IEEE 802.11ad is centered at the 60 GHz radio frequency band and provides transmission bandwidth that is several GHz wide. The receivers designed to process IEEE 802.11ad waveforms employ very high rate analog-to-digital converters, and thus reducing the receiver sampling rate is useful. In a state-of-the-art channel estimation scheme [6], the authors have mitigated the problem of low-rate channel estimation in IEEE 802.11ad by harnessing sparsity in the channel impulse response. They investigated recovery performance through RMSE between the actual and estimated channel. The decrease in RMSE results in a performance improvement at the PHY layer. The effect of this improvement traverses its way up to the vehicular communication stack [7], and leads to a better network performance.

Recently, two approaches of channel estimation have been investigated for the IEEE 802.11p. The first approach demands modification in the structure of the IEEE 802.11p [8,9,10,11,12,13], while the second approach does not require such modification [1,5,13,14,15,16]. In the IEEE 802.11p, channel estimation is performed by transmitting two predefined Long Training Symbols (LTSs) at the beginning of each packet. The channel is then estimated once for each packet, and this estimate is used to equalize the entire packet. IEEE 802.11p does not restrict the packet length. A channel estimate can quickly become less reliable for longer packets in V2V communications. Moreover, the IEEE 802.11p only allows the use of four pilot subcarriers (intended for residual frequency offset correction). These pilot subcarriers are not spaced closely enough to sample the variations of the channel in the frequency domain, which also contributes to performance degradation in V2V communications.

The main motivation behind the proposed scheme is to track the channel variations in time and frequency domain. In [5], authors have investigated that channel estimation in the IEEE 802.11p standard can be improved by exploiting the correlation characteristics between the adjacent symbols of the orthogonal frequency-division multiplexing (OFDM) based data packet via constructing data pilots in the time domain. In this article, we have investigated that the adjacent subcarriers also have strong correlation characteristics in a data packet. We have employed these correlation characteristics in time and frequency domain to construct the reference data pilots for the channel estimation. In this way, an improved channel equalization can be achieved with a little complexity. The main contributions of this paper are listed here:

- We have proposed an end-to-end channel estimation and equalization scheme for the IEEE 802.11p standard. It does not require modifications in the structure of the standard and keeps a balance between computational complexity and BER performance of the overall system.
- In the channel estimation process, we have also utilized the correlation characteristics between adjacent subcarriers in the frequency domain, as well as between adjacent OFDM symbols in time domain.
- The simulation results have demonstrated the performance improvement over CDP and spatial temporal-averaging (STA) schemes for V2V communications.
- We have also presented an intuitive visualization of the V2V channel model which is used in the evaluation of the proposed scheme.

The paper is organized as follows. Section 2 describes the current system model of the IEEE 802.11p transmitter and the channel. Section 3 gives an overview of the current channel estimation schemes for the IEEE 802.11p. Section 4 briefly describes the receiver and the integration of the proposed scheme. Section 5 presents the simulation results and analysis of the proposed iCDP and Section 6 discusses two important issues along with future work suggestions. Finally, Section 7 draws the main conclusions derived from this work.

## 2. System Model of the IEEE 802.11p

### 2.1. OFDM Frame of IEEE 802.11p

**Figure 1:**
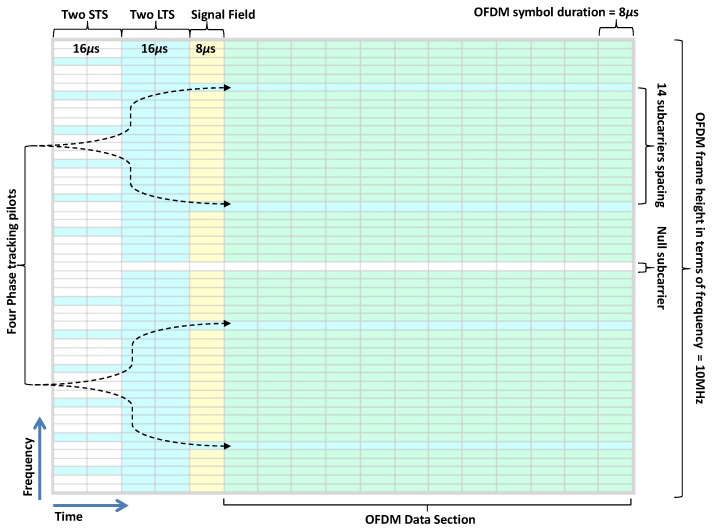
Orthogonal frequency division multiplexing (OFDM) frame format of the IEEE 802.11p.

Figure 1 shows the structure of an OFDM frame of the IEEE 802.11p. The IEEE 802.11p physical layer is nearly the same as IEEE 802.11a. The symbol duration in 802.11p is twice that of 802.11a. The data carried by the closely spaced orthogonal frequency subcarriers helps in improving the spectrum efficiency under harsh conditions of the vehicular data transmission channel. It can support various data rates from 3 to 27 Mbps depending on different modulation and coding schemes, as summarized in Table 1. The Fast Fourier Transform (FFT) size is of 64 points. The packet starts with a preamble that includes Short Training Symbols (STSs) and LTSs, the Signal Field, and the Data Section. The ”Signal Field” provides information related to the type of modulation, coding rate, etc. The Data Section contains information about the transmitted data.

**Figure 2:**
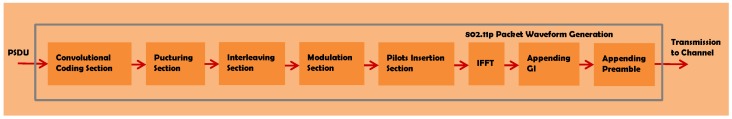
Transmitter block diagram of IEEE 802.11p.

Coarse synchronization is achieved by the ten STSs which are placed at the beginning of each packet. The two LTSs that follow Guard Interval (GI) of STSs are used for the channel estimation and fine synchronization. The GI is placed at this location to cope with the InterSymbol Interference ISI. The Signal Section has only one OFDM symbol. There is no specific limit on the number of OFDM symbols in the Data Section. The block diagram of the IEEE 802.11p transmitter model is shown in Figure 2.

### 2.2. Transmitter Model

The transmitter consists of a convolutional encoder for the forward error correction, a puncturing module for higher data rates, the interleaver to oppose burst errors. Then a modulation module is added for the selection of different schemes. The 4 phase tracking pilots are inserted to the 48 data subcarriers in the pilot insertion module. These phase tracking pilots help in nullifying the phase rotations that are caused by the frequency offsets. Additionally, a total of 11 subcarriers and a null subcarrier is also added to complete a set of 64 subcarriers. After that, an Inverse FFT (IFFT) module is placed to transform the data to the time domain and then a GI and preamble modules are placed to make packet ready for transmission.

### 2.3. Channel Model

Recently, the V2V channel model characteristics have been explored in the literature [17,18,19,20,21,22,23,24,25,26,27,28]. Table 2 summarizes different characteristics of the seven realistic channel models [25,28] of vehicular communication environments. A classical Tapped Delay Line (TDL) channel model, namely, wide stationary uncorrelated scattering (WSSUS), was proposed by [29]. Another closely related channel model is known as non-WSSUS, which is proposed by [25]. These models are accepted as standard channel model for the IEEE 802.11p based systems. These realistic channel models were obtained through a channel measurement campaign which was performed in the metropolitan Atlanta, Georgia, USA. The campaign consisted of six different scenarios. The V2V or (VTV) Expressway Oncoming, the VTV Urban Canyon Oncoming, Roadside To the Vehicle (RTV) Suburban street, the RTV Expressway, the VTV Expressway Same Direction With Wall (SDWW), and the RTV Urban Canyon.

Further experiments were done by the European Telecommunications Standards Institute (ETSI) and a comprehensive vehicular channel model was formulated [28]. This channel model is known as HIgh PERformance radio Local Area Network (HIPERLAN)/2 and it is also available in Matlab 2016 version. This model has a delay profile called Model-E, that represents Non-Line-Of-Sight (NLOS) conditions with an average delay spread of 250 ns. In CDP scheme, the time-variant characteristics of channel models [25] have been employed, however its impact on the overall OFDM performance due to intercarrier interference under HIPERLAN/2 SISO fading channel model [28] still needs further investigations. We have investigated this severe channel model for the proposed channel estimation. We have also tested the proposed scheme for the VTV Express oncoming channel model as shown in Table 2. The time and frequency variations have been analyzed in the paper. We will use a short-term HIPERLAN-E for the HIPERLAN/2 Model-E channel model in this article.

## 3. Overview of Current Channel Estimation Schemes

Recently, a number of channel estimation schemes have been devised for the IEEE 802.11p. The pilot symbols placement in the time-frequency plane is very important for the estimation of the channel. However, the placement of pilots in the current IEEE 802.11p standard appears to be insufficient for tracking under the rapid motion of vehicles. One of the main reasons is that the two LTSs cannot provide enough information in the frequency domain. Two kinds of channel estimation scenarios are discussed in the literature. The first scenario demands modification in the IEEE 802.11p standard and the second scenario does not require such modification. The IEEE 802.11a chips are low cost and readily available in the market. These chips can be modified in a cost-efficient way if we follow the second kind of scenario.

### 3.1. Scenario 1: Modification in IEEE 802.11p

#### 3.1.1. The Midamble Based Channel Estimation Scheme (MBCE)

This work was proposed in [10,11] to mitigate the effects of time-varying channels. The midamble pilots are placed periodically in the data symbols. If the number of these midamble sequences is sufficient, then there is no limit on the length of Data section. At first, the channel estimation is performed based on the two LTS, and then the estimated channel response is used to equalize the next data symbols. Afterward, the midamble pilots are used to update and track the channel, using the same channel estimation procedure as that in the case of LTS. The ratio of the number of data symbols and the number of midambles is called *c*, and the simulation results have shown that a minor value of *c* can effectively enhance the performance. However, improvement in the performance is achieved at the expense of spectrum efficiency, which results in lower transmission rates. In addition to this, the optimized selection of parameter *c* is still not resolved.

#### 3.1.2. Time Domain Least Square Estimation (TDLSE) Scheme

Another scheme known as TDLSE was proposed in [12,13] for the tracking of the time-varying channel. The main idea behind this scheme was reported by [30] and then was further exploited in a different literature [31,32,33]. The authors have proposed to insert a Zadoff-Chu sequence or Pseudo Noise (PN) sequence into the prefix section for further improving the channel estimation. The channel impulse response is initially derived by the Least Square (LS) estimation of the received signal and the transmitted matrix which consists of the replaced prefix, known as Zadoff-Chu sequence. In addition to that, the estimated channel response is averaged with the adjacent symbols. In the end, the LS equalization is performed and the simulation results show that both sequences based TDLSE can achieve significant improvement in the channel estimation as compared to other schemes.

### 3.2. Scenario 2: Remain the Structure of the IEEE 802.11p

**Definition** **1.** LS Estimation: The commonly used channel estimation scheme for the IEEE 802.11p is LS estimation [1]. It combines the two received LTSs, which are represented by $R_{T_{1}}\left(k\right)$ and $R_{T_{2}}\left(k\right)$. Let X(k) represents the transmitted LTS. The channel response can be derived by Equation (1).

(1) $$H\left(k\right)=\frac{R_{T_{1}}\left(k\right)+R_{T_{2}}\left(k\right)}{2X(k)}\;$$

This channel response H(k) can be used for the equalization of the subsequent symbols, with the assumption that the channel will remain constant during the symbol duration. However, the channel response for the IEEE 802.11p does not remain constant for the symbol duration, and LS estimation cannot provide good results in V2V channels.

#### 3.2.1. Wiener Filter (WF) Based Scheme

The LS estimation is not sufficient for the rapidly varying channel conditions. Therefore, a work in [14] proposes a Wiener filter based estimation. The Wiener filter is deployed between the channel estimation and equalization. It searches the optimal coefficients to minimize the mean square error (MSE) of the channel response. The design of the Wiener filter is very important in order to gain channel estimation quality. The Wiener filter is based on three parameters such as, (1) the estimated SNR as described in [34]; (2) maximum excess delay $\tau_{max}$; and (3) the power delay profile (PDP), which is represented as P(τ). In the vehicular time-varying channels, the derivation of these parameters is complex and quite a challenging task, especially the $\tau_{max}$ and P(τ). One simple approximation is to consider the value of $\tau_{max}$ equal to 1.6 μs, which is the time taken by the GI. The shape of PDP can be assumed as rectangular or exponentially decaying. It is observed through the simulation results that the Wiener filter application can achieve better MSE, which eventually improve the packet error rate (PER). However, the assumptions related to the value of $\tau_{max}$ and the shape of PDP can lead to suboptimal performance due to the uncertainties due to these assumptions.

#### 3.2.2. Generalized Discrete Prolate Spheroidal (GDPS) Sequences Based Scheme

Another iterative approach is followed by [15], that estimates the channel based on the generalized DPS sequences. Theoretically, the DPS sequences can tighten the design of subspace and the iterative estimation mechanism provides the pilot information. This concept was investigated in [35]. The DPS sequences minimize the MSE by tightening the subspace. The generalized DPS sequences can simplify the channel estimation and it only requires DSP coefficients. The iterative algorithm requires higher computational complexity and in order to deal with such problem, an enhanced pilot symbol pattern was proposed as well in [35]. The overall kernel is postamble appended after the Data section. However, such reduction in computational complexity will lead to a decrease in the efficiency and incompatibility with the standard.

#### 3.2.3. Spectral-Temporal Averaging (STA) Based Scheme

Another excellent scheme [36] that addresses the challenges of time-varying nature of V2V channel is known as STA. The STA is actually an approach to estimate the dynamic nature of such kind of communication channels. If *i* is the index of OFDM data symbol, then (i−1) th estimated channel response which is represented by $H_{STA,i-1}\left(k\right)$, equalizes the received data symbol $S_{R,i}\left(k\right)$ and given by Equation (2).

(2) $${\hat{S}}_{T,i}\left(k\right)=\frac{S_{R,i}\left(k\right)}{H_{STA,i-1}\left(k\right)}\;$$

Where $H_{STA,0}\left(k\right)$ is the channel response of the 0th data symbol. It is obtained from Equation (1) by using the LTSs. A modulation scheme dependent demmapper transforms ${\hat{S}}_{T,i}\left(k\right)$ to ${\hat{X}}_{i}\left(k\right)$. The phase tracking pilot subcarriers at ${\hat{X}}_{i}\left(k\right)$ with *k* = −21, −7, 7, 21 are also combined with the frequency domain values in the standard. Let $H_{i}\left(k\right)$ be the initial estimate of the channel obtained by Equation (3).

(3) $$H_{i}\left(k\right)=\frac{S_{R,i}\left(k\right)}{{\hat{X}}_{i}\left(k\right)}\;$$

$H_{i}\left(k\right)$ is derived from the knowledge of data symbols, however, these data symbols might be incorrectly mapped especially at the lower Signal-to-Noise Ratio (SNR) conditions. Therefore, [36] considered that averaging in both the frequency and time domains can help in improving accuracy. The averaging in the frequency domain is given by Equation (4).

(4) $$H_{update}\left(k\right)=\sum_{\lambda =-\beta }^{\lambda =\beta }\omega_{\lambda }H_{i}\left(k+\lambda \right)\;$$

The expression 2β+1 represents the subcarriers that are averaged. The set of weighting coefficients $\omega_{\lambda }$ has unit sum and is equal to $\omega_{\lambda }=1/\left(2\beta +1\right)$. The averaging in the time domain is given by Equation (5).

(5) $$H_{STA,i}\left(k\right)=\left(1-\frac{1}{\alpha }\right)H_{STA,i-1}\left(k\right)+\frac{1}{\alpha }H_{update}\left(k\right)$$

The updating parameter α is related to the Doppler spread. The values of α and β parameters are dependent on the types of vehicular channels. Significant accuracy can be achieved by adjusting these values according to the knowledge of the vehicular channel environment. One source of such knowledge is through the Global Positioning System (GPS), as discussed in [36]. However, such a kind of information is practically quite hard to obtain. It is was investigated that fixed values of these parameters can be a good balance of algorithm simplicity and an acceptable performance degradation.

Another channel tracking technique [16] performed the initial channel estimation through the preamble just like the midamble base estimation scheme. The next symbols are then estimated in a specific order, that includes the equalization, decision direct estimation, and smoothing process. The channel tracking algorithm in the decision directed scheme is the same as Equations (2) and (3). However, at lower values of SNR, the wrong decisions can propagate and cause an error in the channel tracking process. Therefore, a smoothing process has been proposed to mitigate the part of estimation noise. The performance improvement is depicted in the simulation results. This smoothing operation brought a huge complexity into the computation process, which is mainly caused by the multiplication of larger size matrices. This computational complexity was reduced by two algorithms, known as lossless complexity reduction and lossy complexity reduction. The lossless complexity reduction deployed the Singular Value Decomposition (SVD) to lower the burden of matrix multiplication. The lossy complexity reduction, on the other hand, ignores some subcarriers to reduce complexity with little degradation in the performance.

#### 3.2.4. Pseudo-Pilot Based Scheme

Besides TDLSE scheme, a pseudo pilot based channel estimation scheme was proposed in [13]. The basic algorithm of the pseudo-pilot based scheme was proposed in [37,38] and the pseudo pilots were assumed as true pilots. This scheme is based on the regression polynomial to estimate the channel response [39]. Further, in [39,40], the least square fitting was investigated to calculate the regression polynomial coefficients. The algorithm of pseudo pilot scheme is based on three steps, such as (1) precalculation; (2) real-time estimation; and (3) channel tracking. The first step needs predefinition of the regression polynomial and the prescoring of the related matrix. The second step at first calculates the channel impulse response on the pilot subcarriers, and then it estimates the complete channel impulse response. Afterward, this complete response is used to derive a better channel response by using a scheme that is similar to the decision directed (DD) scheme. The third and final step is an improvement of the pseudo-pilot algorithms [37,38]. The initial coefficients are derived from the preamble and then the pilots are deployed to track the channel variations in the adjacent symbols with performance improvement in the pseudo-pilot algorithm.

#### 3.2.5. DFT Based Scheme

To obtain a trade-off between performance and complexity in vehicular communication, a Discrete Fourier Transform (DFT) based channel estimation technique was introduced in [41]. In this scheme, the channel response obtained by LS estimation is converted back to the time domain by taking its inverse Fourier transform. In this scheme, the coefficients for the maximum channel delay are selected after ignoring the noise-containing coefficients of the time domain channel response. These selected components are then returned back to the frequency domain and the equalization is performed with the simulation results better than LS estimation. This scheme performs better than LS, STA, and CDP under the conditions when the maximum Doppler shift is under 10 Hz, however, as the Doppler shift increases above 10 Hz, its performance degrades severely.

#### 3.2.6. CDP Based Scheme

In [5], the preamble-based channel response is utilized to construct data pilots in subsequent data symbols. The high correlation characteristics of the channel response between the adjacent data symbols are employed by exploiting the four-phase tracking pilots to achieve better performance than STA. The estimation process in the CDP scheme is composed of five steps, including (1) equalization; (2) constructing data pilot; (3) LS estimation; (4) demapping; and (5) comparison. The first three steps in the CDP scheme are similar to the STA scheme. In the next two steps, the high correlation characteristic is utilized to achieve performance improvement over STA and decision directed schemes, especially in the higher SNR region.

Table 3 presents the comparison of the discussed schemes. The Computational Complexity Level is represented by CCL, and the BER Performance Level is represented by PL. CCL 0.1 means very low computational complexity (e.g., LS) and CCL 0.5 means very high computational complexity (e.g., GDPS and DD). Please note that this grading is for understanding the difference between the computational complexities of various schemes. PL 0.1 means excellent performance and 0.5 means a severe performance degradation (e.g., LS). The terms SM and CSI denote System Modification and Channel State Information respectively in Table 3.

## 4. The Proposed Scheme

**Figure 3:**
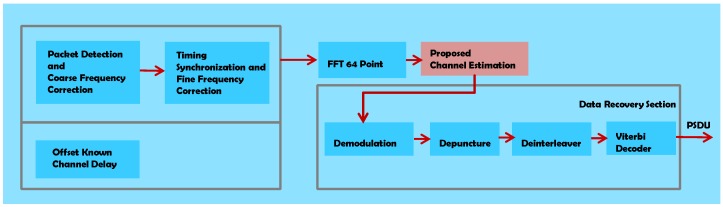
IEEE 802.11p receiver block diagram.

The block diagram of the receiver is shown in Figure 3. The pink shaded box depicts the proposed channel estimator. It is placed after the 64 point FFT block. Table 4 presents the timing parameters used in the simulation of the proposed scheme, and they are in accordance with Section 18 of the IEEE standard 802.11p [1].

The timing synchronization and fine frequency correction are performed based on the output of packet detection and coarse frequency correction block. The detail about this block can be found in [42].

**Definition** **2.** Packet Detection: The process of detecting a packet based on the information in the received time domain signal $X_{t}$, such as channel bandwidth. The output of this process is the offset, from the start of the received input waveform to the start of the detected preamble using auto-correlation.

**Figure 4:**
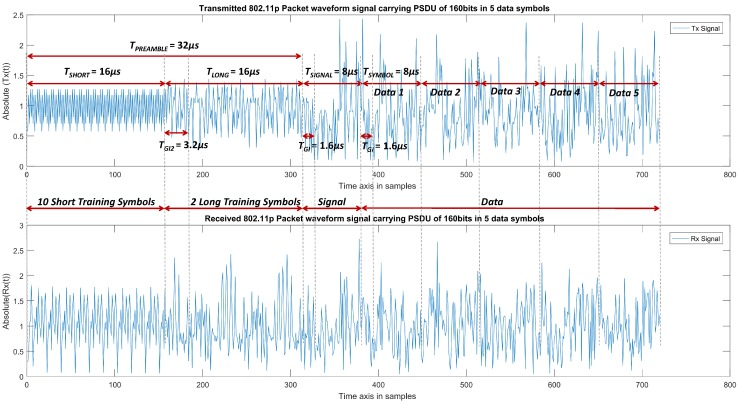
Transmitted and Received IEEE 802.11p packet.

Figure 4 shows a single IEEE 802.11p packet waveform, where the upper part is the transmitted time domain signal and the lower part is the received time domain signal ready for channel estimation block. It should be noted that the transmitted time domain waveform $T_{x}\left(t\right)$ is a vector composed of complex numbers, therefore we have taken the absolute of the transmitted waveform i.e., $\sqrt{T_{x}{\left(t\right)}^{2}}$ and plotted in the upper part of Figure 4.

**Definition** **3.** Coarse Frequency correction: It is the correction in terms of frequency error which is estimated by utilizing the pre-known STS training field. The estimate contains the carrier frequency offset in Hertz. The short length of the periodic sequence in the STS allows coarse frequency offset estimation.

Similarly, the received waveform signal $R_{x}\left(t\right)$ that is modulated by channel [28] and at SNR=35 dB is drawn in the lower part of Figure 4 as $\sqrt{R_{x}{\left(t\right)}^{2}}$. For the purpose of clarity, we have plotted only 5 data symbols. Figure 4 also gives the detailed timing information in terms of parameters that are described in Table 4. The output of the FFT block is $S_{R,i}\left(k\right)$, which is the frequency domain representation of the received symbol at *k*th subcarrier. In contrast to the CDP scheme, the proposed scheme also includes the 4 phase tracking pilots and therefore *k* represents 52 subcarriers.

The proposed estimator updates the *i*th symbol’s channel estimate by using the channel response $H_{CDP,i-1}\left(k\right)$, obtained from the previous (i−1)th symbol. The equalization process is given by Equation (6).

(6) $${\hat{S}}_{T,i}\left(k\right)=\frac{S_{R,i}\left(k\right)}{H_{CDP,i-1}\left(k\right)}\;$$

**Figure 5:**
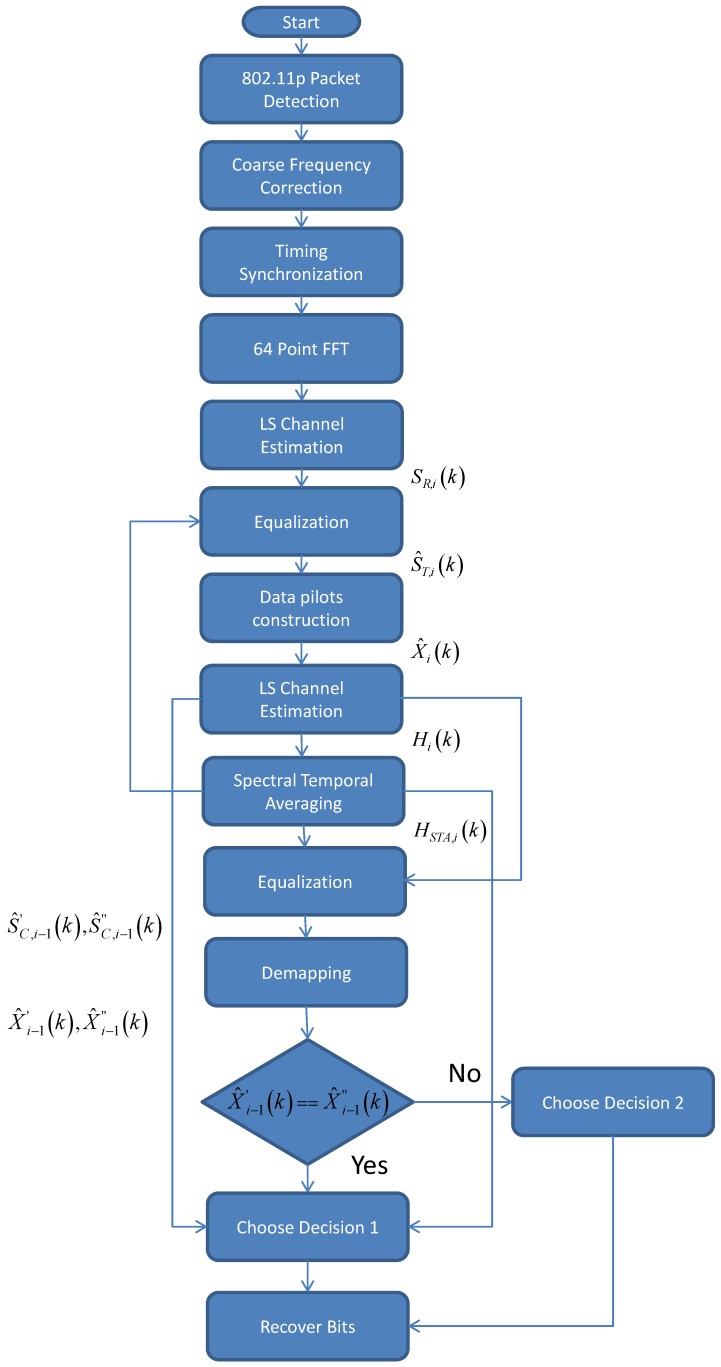
Process flow of the proposed algorithm.

Since ${\hat{S}}_{T,i}\left(k\right)$ contains a total of 52 subcarriers. In order to construct 52 pilots, the ${\hat{S}}_{T,i}\left(k\right)$ is divided into two groups of subcarriers, i.e., the data subcarriers group ${\hat{S}}_{T,i}\left(k_{D}\right)$ and the phase tracking pilots subgroup ${\hat{S}}_{T,i}\left(k_{P}\right)$. These groups are then demodulated separately and then demapped to the respective constellation points to generate the two groups of pilots, i.e., one for the data subcarriers and the other for the phase tracking pilots. The reason behind the construction of the phase tracking pilots is to get more information for the comparison block. The process flow chart of the proposed channel estimator algorithm is shown in Figure 5. It is clear from the process flowchart in Figure 5 that the time domain signal in Figure 4 is now converted to the frequency domain by taking the 64 points FFT. Then the LS estimation is performed on this signal using Equation (1). As stated earlier, the next task is to perform the equalization to get a rough estimate of the transmitted LTS. The initial Data pilots are created and then again LS estimation is performed which is based on the constructed data pilots.

Since the adjacent subcarriers to the phase tracking pilots have a higher correlation with them, so the constructed phase tracking pilots and the originally known phase tracking pilots are compared to decide the channel response in those regions, which helps in improving the accuracy of the overall system. In the CDP estimation scheme, the assistance of demapping is used to partially alleviate the impact of noise from the other interferences. Then, the remaining error is further reduced by exploiting the correlation characteristics of the channel for the two adjacent symbols. The constructed data pilot ${\hat{X}}_{i}\left(k\right)$ in [5] are then employed to obtain the *i*th data symbol’s channel response by using the Equation (3). We further investigated that even though $H_{i}\left(k\right)$ is a relatively accurate estimate of the channel response, however, it is can be improved by exploiting the high correlation characteristics of the channel. Therefore, $H_{i}\left(k\right)$ is used to equalize $S_{R,i-1}\left(k\right)$ in Equation (7).

(7) $${\hat{S^{\prime }}}_{C,i-1}\left(k\right)=\frac{S_{R,i-1}\left(k\right)}{H_{i}\left(k\right)}$$

Again, the $S_{R,i-1}\left(k\right)$ is then equalized by $H_{CDP,i-1}\left(k\right)$, i.e., the previous symbol’s estimated CR, which has been used before in Equation (6). In order to make a comparison, $S_{R,i-1}\left(k\right)$ is also equalized by the $H_{CDP,i-1}\left(k\right)$ and the resulting equalized ${\hat{S^{\prime \prime }}}_{C,i-1}\left(k\right)$ is given by Equation (8).

(8) $${\hat{S^{\prime \prime }}}_{C,i-1}\left(k\right)=\frac{S_{R,i-1}\left(k\right)}{H_{CDP,i-1}\left(k\right)}$$

The one to one comparison of ${\hat{S^{\prime }}}_{C,i-1}\left(k\right)$ and ${\hat{S^{\prime \prime }}}_{C,i-1}\left(k\right)$ is made by appropriately demapping them to their respective constellation points ${\hat{X^{\prime }}}_{i}\left(k\right)$ and ${\hat{X^{\prime \prime }}}_{i}\left(k\right)$. The proposed demapping process starts by using constellation demodulation of ${\hat{S^{\prime }}}_{C,i-1}\left(k\right)$ and ${\hat{S^{\prime \prime }}}_{C,i-1}\left(k\right)$, using the information from the standard IEEE 802.11 modulation coding scheme (MCS) table. Let us take the case of mcs = 2, so that after the demodulation the ${\hat{S^{\prime }}}_{C,i-1}\left(k\right)$ is now represented by ${\hat{S^{\prime }}}_{Cdn,i-1}\left(k\right)$ and ${\hat{S^{\prime \prime }}}_{C,i-1}\left(k\right)$ is represented by ${\hat{S^{\prime \prime }}}_{Cdn,i-1}\left(k\right)$. For this particular configuration, ${\hat{S^{\prime }}}_{Cdn,i-1}\left(k\right)$ can take four different values represented by (n=1,2,3,4) Equation (9) to Equation (12).

(9) $${\hat{S^{\prime }}}_{Cd1,i-1}\left(k\right)\approx \frac{1}{\sqrt{2}}+\frac{i}{\sqrt{2}}\;$$

(10) $${\hat{S^{\prime }}}_{Cd2,i-1}\left(k\right)\approx \frac{1}{\sqrt{2}}-\frac{i}{\sqrt{2}}\;$$

(11) $${\hat{S^{\prime }}}_{Cd3,i-1}\left(k\right)\approx -\frac{1}{\sqrt{2}}+\frac{i}{\sqrt{2}}\;$$

(12) $${\hat{S^{\prime }}}_{Cd4,i-1}\left(k\right)\approx -\frac{1}{\sqrt{2}}-\frac{i}{\sqrt{2}}\;$$

Let say P1, P2, P3, and P4 represent the values $\frac{1}{\sqrt{2}}+\frac{i}{\sqrt{2}}$, $\frac{1}{\sqrt{2}}-\frac{i}{\sqrt{2}}$, $-\frac{1}{\sqrt{2}}+\frac{i}{\sqrt{2}}$, and $-\frac{1}{\sqrt{2}}-\frac{i}{\sqrt{2}}$ respectively. In order to choose the best value, we need to take the absolute difference of ${\hat{S^{\prime }}}_{Cdn,i-1}\left(k\right)$ (where *n* can take any value from 1 to 4) from P1, P2, P3, and P4 to get G1, G2, G3, and G4 as shown in Equation (13).

(13) $$Gn=abs({\hat{S^{\prime }}}_{Cdn,i-1}\left(k\right)-Pn)\forall n\;$$

Then the argument or index of the minimum value from G1,G2,G3,G4 is selected as shown in Equation (14).

(14) index=argmin(G1,G2,G3,G4)

The prediction of the actual value of ${\hat{X^{\prime }}}_{i}\left(k\right)$ and similarly that of ${\hat{X^{\prime \prime }}}_{i}\left(k\right)$ can be found from Equation (15).

(15) $${\hat{X^{\prime }}}_{i}\left(k\right)=Pn,if\left\{index==n\right\}\forall n\;$$

If ${\hat{X^{\prime }}}_{i}\left(k\right)\neq {\hat{X^{\prime \prime }}}_{i}\left(k\right)$, it means that the kth subcarrier’s ${\hat{X^{\prime }}}_{i}\left(k\right)$ is not valid and the assumption is made that $H_{CDP,i}\left(k\right)=H_{CDP,i-1}\left(k\right)$. Additionally, if the ${\hat{X^{\prime }}}_{i-1}\left(k\right)={\hat{X^{\prime \prime }}}_{i}\left(k\right)$, then it can be assumed that $H_{CDP,i}\left(k\right)=H_{i}\left(k\right)$, which is indicated as Decision 1 in Figure 5. We argue that the two adjacent data symbols have high correlation in frequency as well. Therefore if ${\hat{X^{\prime }}}_{i}\left(k\right)\neq {\hat{X^{\prime \prime }}}_{i}\left(k\right)$, it indicates that the *k*th subcarrier’s ${\hat{X^{\prime }}}_{i}\left(k\right)$, which demapped after Equation (6) is incorrect and we should define that $H_{iCDP,i}\left(k\right)=H_{iCDP,i-1}\left(k\right)$, i.e., the previous symbol’s estimated CR. It is indicated as Decision 2 in Figure 5. Moreover, in contrast to the CDP scheme where otherwise, if ${\hat{X^{\prime }}}_{i-1}\left(k\right)={\hat{X^{\prime \prime }}}_{i}\left(k\right)$ then $H_{CDP,i}\left(k\right)=H_{i}\left(k\right)$. We argue that the superior performance over CDP scheme can be obtained by taking into consideration $H_{i}\left(k\right)$ obtained previously from Equations (3) and (5) through the Equation (16).

(16) $$H_{iCDP,i}\left(k\right)=\frac{\frac{1}{2}H_{STA,i-1}\left(k\right)+\frac{1}{2}H_{update}\left(k\right)+H_{i}\left(k\right)}{2}$$

where $H_{iCDP,i}\left(k\right)$ is the proposed channel estimate. The next section evaluates the proposed estimator over the severe channel conditions of [28] and also provides comparisons with the closely related schemes. Let us assume that $|H_{\left(i,k\right)}|$ represents the absolute of the estimated channel.

## 5. Simulation Results and Analysis

**Figure 6:**
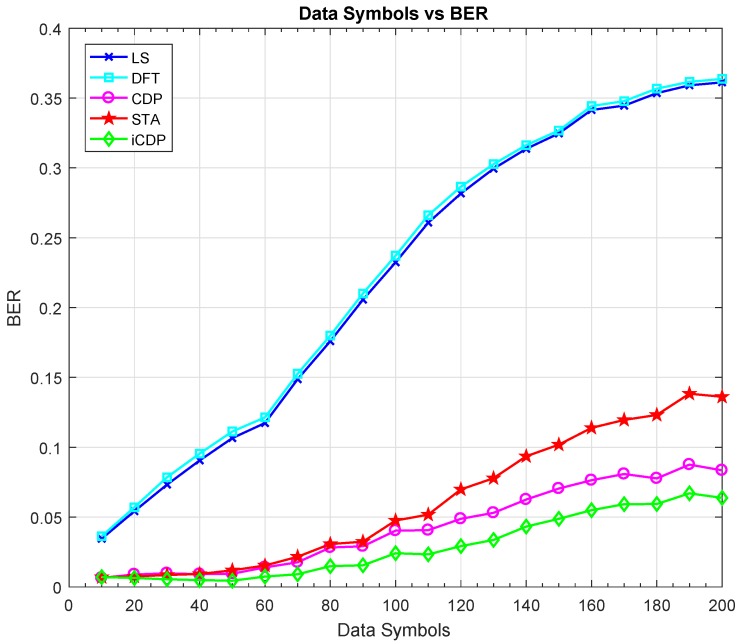
Bit Error Rate (BER) vs. length of data symbols for HIgh PERformance radio Local Area Network (HIPERLAN-E) Channel and $f_{d}$ = 1200 Hz.

The simulation platform used in this article is Matlab 2016b. The BER simulation results of different configurations are presented in this section. We have compared the proposed iCDP scheme with LS, DFT, STA, and CDP schemes. The value of parameters α and β is set to 2 for the optimal performance of the STA scheme as discussed in [36]. The performance of IEEE 802.11p is analyzed under the different length of packets i.e., from 10, 20, 30, and up to 200 OFDM data symbols. The simulation results reveal that LS and DFT channel estimation schemes have higher BER values, whereas STA, CDP, and iCDP are comparatively better under all symbol lengths as shown in Figure 6.

**Figure 7:**
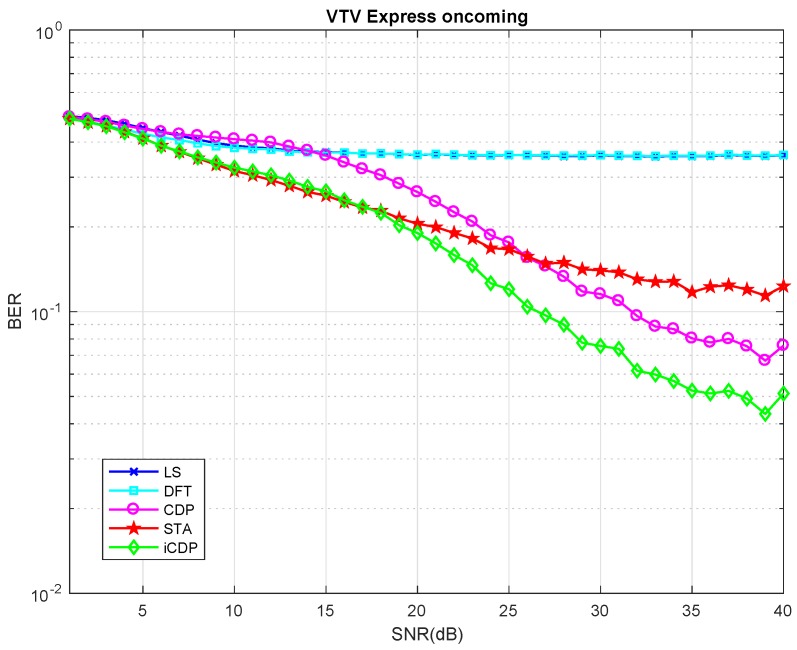
Vehicle-to-Vehicle (VTV) Express oncoming Channel, Data Symbols = 100.

The value of maximum Doppler shift is set to 1200 Hz for the HIPERLAN-E channel and the modulation coding scheme is mcs=2. It is also obvious from Figure 6 that the BER values for STA, CDP, and iCDP remain almost same for the 10 and 20 data symbol lengths, however, as the length increases, iCDP tends to perform better than STA and CDP. The next test is performed for the VTV express oncoming channel model as shown in Figure 7. It is also clear from the results that under the lower SNR regions the STA scheme performs better than CDP scheme, however, the proposed iCDP scheme performs better than CDP scheme and nearly similar to the STA scheme.

In the higher SNR regime, the CDP scheme outperforms the STA scheme. This is due to the fact that under the lower SNR the noise and interferences are strong enough to move the ${\hat{S}}_{T,i}\left(k\right)$ to the incorrect locations, which results in demapping of ${\hat{X}}_{i}\left(k\right)$ to the incorrect constellation points.

The increase in SNR reduces these incorrect demappings and the CDP scheme performs better in this regime. In contrast to the CDP scheme, the iCDP is inherently SNR aware. This is due to the reason that the construction of data pilots is followed by LS channel Estimation and STA equalization, which reduces the error demapping probability of ${\hat{X}}_{i}\left(k\right)$ to the constellation points. The results show that the STA and iCDP schemes perform equally well as compared to CDP up to the SNR value of 18 dB. On the other hand, CDP and iCDP perform better than STA from the SNR greater than 26 dB. The iCDP overall performs better than the CDP scheme. Due to the page limit, the result of the rest of the 5 channel models are skipped and the next results are for the HIPERLAN-E channel model.

**Figure 8:**
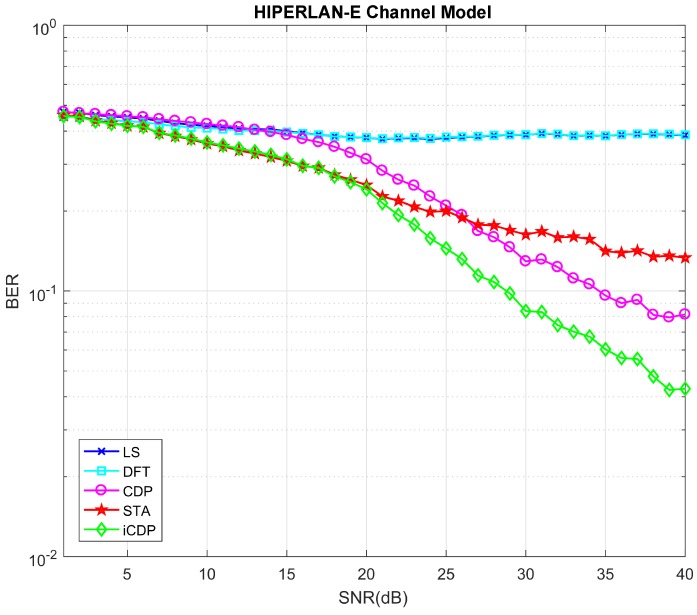
HIPERLAN-E channel model with Data Symbols = 100.

Under urban traffic scenario the number of buildings and vehicles increases, hence scattering and reflection of the transmitted signals also increase. This phenomenon leads to an increase in the number of multi-path fading. The HIPERLAN-E channel model has a total of 18 multi-paths. Figure 8 shows the result of the BER performance curves of different schemes for this channel model. The results show that the STA and iCDP schemes perform equally well as compare to CDP up to the SNR value of 20 dB. On the other hand, CDP and iCDP perform better than STA from the SNR greater than 22 dB.

**Figure 9:**
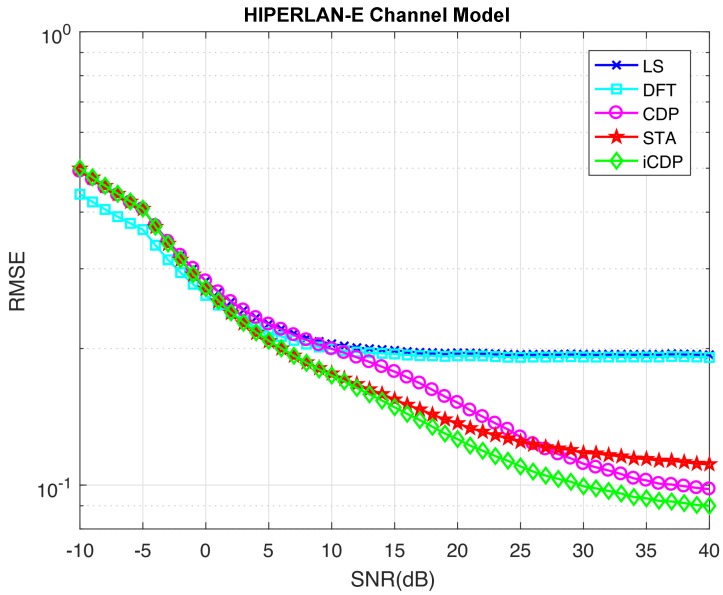
Root-mean-square error (RMSE) comparison for HIPERLAN-E channel model with Data Symbols = 200.

The performance comparison in terms of RMSE is shown in Figure 9. The method to obtain RSME is described in [6]. It is clear that at an SNR of −10 dB, the DFT scheme performs better than others. The RMSE value DFT scheme is 0.4381 at −10 dB, while the other schemes have roughly the same value of 0.4994. It is due to the fact that DFT ignores the noise-containing coefficients of the time domain channel response. However, the RMSE performance of DFT degrades as the SNR becomes bigger. The iCDP and STA schemes perform almost similar till the value of SNR equal to 8 dB. However, CDP scheme performs similarly to LS scheme until SNR equal to 8 dB. The RMSE values obtained at SNR equal to 8 dB for LS, DFT, CDP, STA, and iCDP are: 0.2111, 0.2046, 0.209, 0.1865, and 0.1860 respectively. If we go on further at the point where SNR is 26 dB, then the STA and CDP schemes have roughly similar RMSE value of 0.1227 and the iCDP scheme has a value of 0.1071. The RMSE values of DFT and LS schemes are 0.1916 and 0.1943 respectively. Finally, at an SNR of 40 dB, the RSME value of iCDP is 0.0899 and corresponding RMSE values for CDP and STA are 0.9796 and 0.1112 respectively. The RMSE for LS and DFT seems saturated at roughly 0.1916 with no further improvement in performance. This result demonstrates the performance improvement of the proposed scheme over DFT/LS, STA, and CDP schemes with the RMSE percentage differences of 72.3%, 21.1%, and 8.5% respectively at SNR of 40 dB.

**Figure 10:**
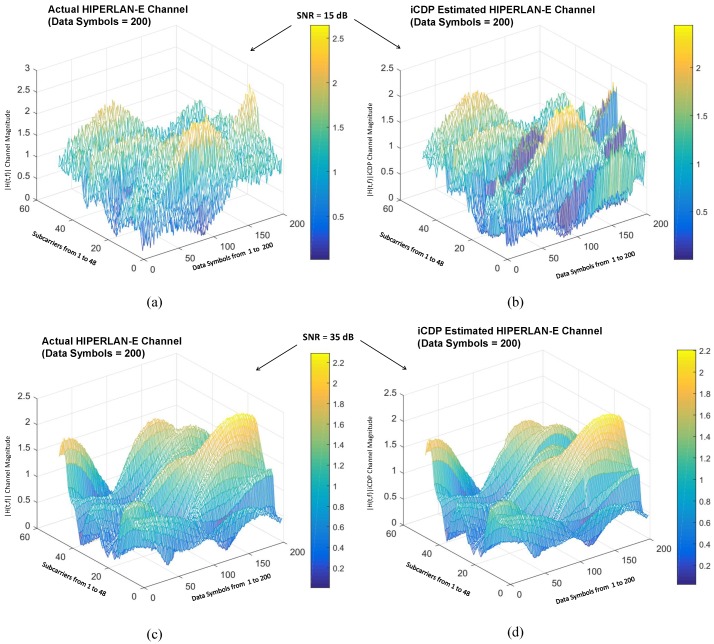
Actual and Estimated HIPERLAN-E channels: (**a**) Actual channel at SNR = 15 dB. (**b**) Estimated channel at SNR = 15 dB. (**c**) Actual channel at SNR = 35 dB. (**d**) Estimated channel at Signal-to-Noise Ratio (SNR) = 35 dB.

It is equally important to visualize the 3-dimensional image of the actual HIPERLAN-E channel and its estimation by the proposed iCDP scheme to show that it tracks the channel in time and frequency domain for the different SNR values. Figure 10 shows the effect of lower and higher SNR on the HIPERLAN-E channel model. Figure 10a shows the actual channel conditions at under $f_{d}=1200$, data symbols = 200, and SNR = 15 dB. Figure 10b shows the estimation of the actual channel in Figure 10a through the proposed scheme. On the other hand, Figure 10c shows another channel conditions at SNR = 35 dB and Figure 10d shows its estimation through the proposed scheme. It is evident from Figure 9 that at lower SNR, the correlation among the data symbols and frequency subcarriers suffers due to the randomness of the noise. The proposed scheme performs relatively better than CDP under lower SNRs because it constructs the data pilots after taking into consideration the front and previous data symbol in time domain and front and previous frequency carrier in the frequency domain.

**Figure 11:**
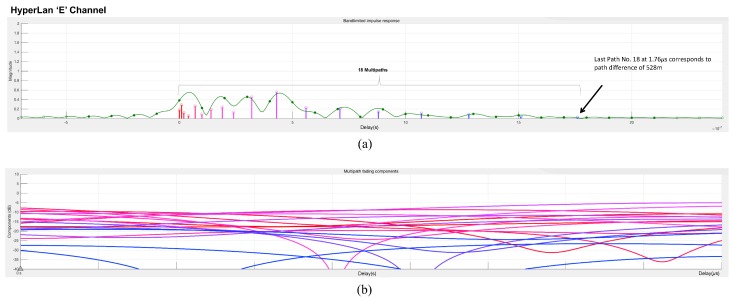
Multipath components of the HIPERLAN-E channel: (**a**) Bandlimited impulse response. (**b**) Multipath fading components after 850 μs.

Figure 11 shows the multipath components of the HIPERLAN-E channel used in the simulation of the proposed scheme. The bandlimited impulse response in Figure 11a shows 18 different paths that are arriving at the receiver vehicle with different amplitudes and delays. Figure 11b shows the contribution of these components with the passage of time up to 850 μs. The delay of the first path is set to 0 μs. For subsequent paths, a 1 μs delay corresponds to a 300 m difference in path length. In vehicular communication, the multipath environments have reflected paths that can be up to several kilometers longer than the shortest path. With the path delays specified above, the last path is 528 m longer than the shortest path, and thus arrives 1.76 μs later which is also obvious from the specifications in Table 2. The path delays and path gains specify the channel’s average delay profile.

Typically, the average path gains decay exponentially with delay (i.e., the dB values decay linearly), but the specific delay profile depends on the propagation environment. In the delay profile specified in Figure 11, we assumed the specifications of Table 2 HIPERLAN-E channel model.

### Computational Complexity

**Figure 12:**
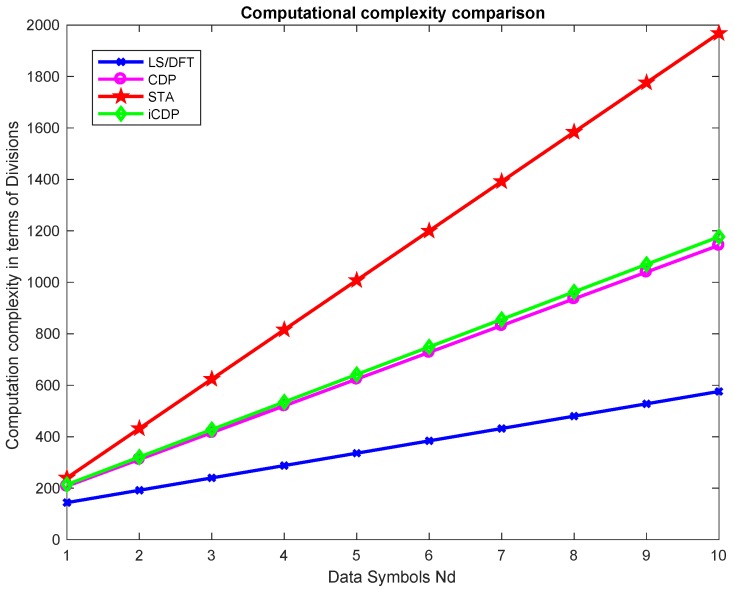
Computation complexity comparison of different schemes.

Figure 12 shows the comparison of computation complexity in terms of the number of additions, as a function of the number of data symbols $N_{d}$. Table 5 summarizes the comparison of the computation complexity of different scheme in terms of divisions, multiplications, additions, and subtractions.

**Figure 13:**
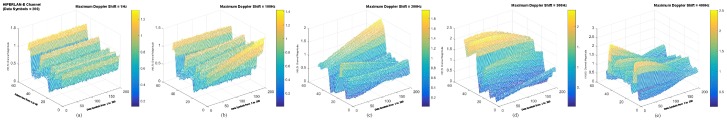
Impact of $f_{d}$ on the channel conditions. (**a**) $f_{d}$ = 1 Hz, (**b**) $f_{d}$ = 100 Hz, (**c**) $f_{d}$ = 200 Hz, (**d**) $f_{d}$ = 300 Hz, and (**e**) $f_{d}$ = 400 Hz.

The higher Doppler shift causes stronger variation in the time domain as well. It is therefore essential to visualize how the value of $f_{d}$ effects the channel conditions in time domain or along the length of data symbols. Figure 13 shows this effect for different values of $f_{d}$ i.e., 1, 100, 200, 300, and 400 Hz.

The impact of these values on the channel conditions is shown in Figure 13a–e. It is interesting to notice that at a very low value of $f_{d}$ = 1 Hz, the channel remains almost invariant in the time axis. Then the variations become larger as the value of $f_{d}$ increases.

## 6. Issues and Future Work

There are two main issues of the discussion and future work. The first issue is related to the computational complexity of the channel estimation process. The schemes that follow scenario 1 achieve better BER curves at the expense of the computation complexity, which is the outcome of complex matrix multiplications. The higher computational complexity leads to a higher requirement of hardware resources, which ultimately requires more energy. This energy demand becomes a critical issue for electric vehicles as discussed in [43]. The proposed scheme keeps the requirement of hardware resources low. However, if computational complexity is not the major concern then the proposed scheme can be further improved by constructing data pilots using Deep Learning techniques.

The second issue is related to the proper utilization of the α and β parameters, which represent the time and frequency variations. Most of the current schemes utilize these parameters, with the assumption of prior knowledge. However, those schemes not mentioned the determination process of these parameters. Some authors [36] determined these parameters from a difficult realization of map knowledge and GPS. However, due to mobility and highly random road scattering variants, these parameters are hard to determine. One future consideration is to devise an algorithm to determine or predict these parameters or lower their dependence on the overall system performance.

## 7. Conclusions

This article presents an improved CDP channel estimation scheme for vehicular communications. The vehicular channel is dynamic in terms of frequency and time. An overview of the state-of-the-art vehicular channel estimation schemes is presented briefly in this article. Even though these schemes perform considerably well, however, there are still limitations and challenges. We have discussed these challenges and proposed a scheme which constructs improved pilots from the data and phase tracking symbols through the exploitation of correlation characteristics of vehicular channels. The proposed scheme keeps the structure of IEEE 802.11p physical layer standard intact independent to the nature of the channel. This makes the proposed scheme a suitable candidate for the IEEE 802.11p and different vehicular channel models. The performance evaluation shows that it outperforms the related schemes e.g., CDP scheme, STA scheme, LS scheme, and DFT scheme both in the lower and high SNR regions. In addition to that, the proposed scheme has the little computational complexity as compared to the various V2V channel estimation schemes, e.g., decision-directed scheme, Pseudo pilot scheme, and generalized DPS based scheme.

## 8. Materials and Methods

The source code used in this article will be made available on github. We have used Matlab version 2016b. The CPU used is Intel’s core i7.

## Figures and Tables

**Table 1:** IEEE 802.11p standard parameters.

Parameter	Values
FFT size	64
FFT period	6.4 μs
Symbol duration	8.0 μs
GI duration	1.6 μs
Total subcarriers	52
Pilot subcarriers	4
Data subcarriers	48
Code Rate	1/2,2/3,3/4
Modulation Schemes	BPSK, QPSK, 16QAM, 64QAM
Bit Rate	3, 4.5, 6, 9, 12, 18, 24, 27
Frequency spacing of subcarriers	156.25 KHz
Error correction coding	k = 7 (64 states) convolution code
Bandwidth	10 MHz

**Table 2:** Characteristics of the vehicular channel models.

Channel Model	Doppler Shift (Hz)	Multipaths	Velocity (km/h)	Average Path Gains (dB)	Path Delays (ns)
VTV Express Oncoming	1000–1200	11	104	[0, 0, 0, −6.3, −6.3, −25.1, −25.1, −25.1, −22.7, −22.7, −22.7]	[0, 1, 2, 100, 101, 200, 201, 202, 300, 301, 302]
RTV Expressway	600–700	12	104	[0, 0, 0, −9.3, −9.3, −9.3, −20.3, −20.3, −21.3, −21.3, −28.8, −28.8]	[0, 1, 2, 100, 101, 102, 200, 201, 300, 301, 400, 401]
VTV Express SDWW	900–1150	12	104	[0, 0, −11.2, −11.2, −19, −21.9, −25.3, −25.3, −24.4, −28.0, −26.1, −26.1]	[0, 1, 100, 101, 200, 300, 400, 401, 500, 600, 700, 701]
RTV Urban Canyon	300	12	32–48	[0, 0, 0, −11.5, −11.5, −11.5, −19.0, −19.0, −25.6, −25.6, −28.1, −28.1]	[0, 1, 2, 100, 101, 102, 200, 201, 300, 301, 500, 501]
RTV Suburban street	300–500	12	32–48	[0, 0, −9.3, −9.3, −14, −14, −18, −18, −19.4, −24.9, −27.5, −29.8]	[0, 1, 100, 101, 200, 201, 300, 301, 400, 500, 600, 700]
VTV Urban Canyon Oncoming	400–500	12	32–48	[0, 0, −10, −10, −10, −17.8, −17.8, −17.8, −21.1, −21.1, −26.3, −26.3]	[0, 1, 100, 101, 102, 200, 201, 202, 300, 301, 400, 401]
HIPERLAN−E	1000–1200	18	109.83	[−4.9, −5.1, −5.2, −0.8, −1.3, −1.9, −0.3, −1.2, −2.1, 0.0, −1.9, −2.8, −5.4, −7.3, −10.6, −13.4, −17.4, −20.9]	[0, 10, 20, 40, 70, 100, 140, 190, 240, 320, 430, 560, 710, 880, 1070, 1280, 1510, 1760]

**Table 3:** Comparison of the proposed scheme with existing schemes for the Vehicle-to-Vehicle (V2V) channel Estimation.

Scheme	SM Requirement	CSI Requirement	CCL	PL	Compatibility
STA	No	Yes	0.3	0.2	Invariant
MBCE	Yes	No	0.2	0.1–0.3	Reduced
TDLSE	Yes	No	0.2	0.2	Reduced
LS	No	No	0.1	0.5	Invariant
WF	No	Yes	0.3	0.3	Invariant
GDPS	No	Yes	0.5	0.3	Invariant
Improved GDPS	Yes	Yes	0.4	0.3	Invariant
DD	No	Yes	0.5	0.3	Invariant
DD Lossless reduction	No	Yes	0.4	0.3	Invariant
DD Lossy reduction	No	Yes	0.5	0.3–0.4	Invariant
Pseudo-pilot	No	Yes	0.5	0.3–0.4	Invariant
CDP	No	Yes	0.2	0.1–0.2	Invariant
Proposed	No	Yes	0.3	0.1	Invariant

**Table 4:** Timing parameters used in the proposed scheme.

Parameter	Value	10 MHz Channel Bandwidth
$N_{SD}$ Number of data subcarriers	48	48
$N_{SP}$ Number of pilot subcarriers	4	4
$N_{ST}$ Number of total subcarriers	$N_{SD}\;$+$\;N_{SP}$	52
$\Delta_{F}$ Subcarrier frequency spacing	10 MHz/64	0.15625 MHz
$T_{FFT}$: FFT, IFFT period	1/$\Delta_{F}$	6.4 μs
$T_{GI}$: GI duration	$T_{FFT}$ / 4	1.6 μs
$T_{SIGNAL}$: Duration of the Signal section	$T_{FFT}\;$+$\;T_{GI}$	8 μs
$T_{GI2}$: Training symbol GI duration	$T_{FFT}$ / 2	3.2 μs
$T_{SYM}$: Symbol duration	$T_{FFT}$ + $T_{GI}$	8 μs
$T_{SHORT}$: STS duration	10 × $T_{FFT}$ / 4	16 μs
$T_{LONG}$: STS duration	2 × $T_{FFT}\;$+$\;T_{GI2}$	16 μs
$T_{PREAMBLE}$: Duration of PLCP preamble	$T_{SHORT}\;$+$\;T_{LONG}$	32 μs

**Table 5:** Computation complexity comparison of the schemes.

Scheme	Divisions	Multiplications	Additions	Subtractions
LS	48$N_{d}$ + 96	-	48	-
DFT	48$N_{d}$ + 96	-	48	-
STA	104$N_{d}$ + 104	148$N_{d}$ − 2β$N_{d}$	52 + 2(β + 1)(52 − 2β)$N_{d}$	48$N_{d}$
CDP	192$N_{d}$ + 48	-	48	-
iCDP	104$N_{d}$ + 104	144$N_{d}$	52+240$N_{d}$	48$N_{d}$

## Abbreviations

The following abbreviations are used in this manuscript:

BER	Bit Error Rate
CDP	Constructed data pilots
DFT	Discrete Fourier Transform
DPS	Generalized Discrete Prolate Spheroidal
DSRC	Dedicated Short Range Communications
GI	Guard Interval
iCDP	Improved constructed data piloted
LS	Least square estimation
LTS	Long Training Symbols
MBCE	The Midamble Based Channel Estimation Scheme
MCS	Modulation Coding Scheme
NLOS	Non line of sight
OFDM	Orthogonal frequency division multiplexing
PHY	Physical
PLCP	Physical layer convergence procedure
STS	Short Training Symbols
STA	Spatial temporal Averaging
TDL	Tapped Delay Line
TDLSE	Time Domain Least Square Estimation Scheme
VTV	Vehicle to vehicle
WSSUS	Wide sense stationary uncorrelated scattering
